# Geographic Differences in Obesogenic Behaviors and Overweight and Obesity Among Children and Adolescents

**DOI:** 10.5888/pcd22.250215

**Published:** 2025-10-02

**Authors:** Elizabeth Crouch, Nicholas Yell, Emma Boswell, Cassie Odahowski, Peiyin Hung, Gabriel Benavidez

**Affiliations:** 1 Rural Health Research Center, University of South Carolina, Columbia, South Carolina; 2 Department of Health Services Policy & Management, University of South Carolina, Columbia, South Carolina; 3 Department of Public Health, Baylor University, Waco, Texas

## Abstract

**Introduction:**

Geographic disparities in childhood obesity have been demonstrated, but research examining differences in obesogenic behaviors by rurality is limited. This study examined children’s rates of overweight and obesity, food security, and obesogenic behaviors (ie, physical activity, sleep time, and screen time) by rurality and the association between rurality and these 5 outcomes.

**Methods:**

We analyzed cross-sectional data on children and adolescents aged 10 to 17 years (N = 35,963) from the 2021–2022 National Survey of Children’s Health, a nationally representative sample of children and adolescents. Frequencies, proportions, and unadjusted results were calculated by using descriptive statistics and bivariate analyses. Multivariable logistic regression models were used to study the relationship between rurality and the 5 outcomes.

**Results:**

Compared with urban children, rural children had higher odds of being overweight or obese (adjusted odds ratio [AOR] = 1.36; 95% CI, 1.19–1.56), being food insecure (AOR = 1.27; 95% CI, 1.11–1.44), and not meeting the recommended hours of sleep (AOR = 1.23; 95% CI, 1.07–1.40); and they had lower odds of being physically inactive (AOR = 0.79; 95% CI, 0.70–0.90) and having more than 2 hours of screen time on an average day (AOR = 0.85; 95% CI, 0.75–0.96).

**Conclusion:**

The findings from this study are instructive for community partners and program developers in creating policies and programs that allow for multitiered interventions to promote positive health behaviors to reduce overweight and obesity among all children and adolescents.

**Summary**

**What is already known on this topic?**

Children from rural areas are more likely to be overweight or obese than children from urban areas. Recent research examining rural–urban differences in overweight and obesity, physical activity, and food security among children has not examined screen time or sleep guidelines.

**What is added by this report?**

This is the first study, to our knowledge, to examine geographic differences in the prevalence of overweight and obesity, food security, and obesogenic behaviors (ie, physical activity, sleep time, and screen time), using a national sample of children and adolescents.

**What are the implications for public health practice?**

Findings should be useful for program developers as interventions are designed to improve children’s health behaviors, particularly around sleep and sedentary behavior, in the US.

## Introduction

Health during childhood is strongly associated with health during adolescence and into adulthood (1), making it critical that interventions and programs prioritize children’s health (2). The lasting effect of childhood health has been demonstrated for conditions such as obesity and poor mental health (3,4), yet many chronic conditions, such as obesity, are increasing in the US (5).

The prevalence of childhood obesity has increased in the US, from an estimated prevalence of 14.7% in 1999 to 19.2% in 2018 (6). The most recent data put the proportion of children and adolescents with obesity at nearly 20%, with one of the Healthy People 2030 goals of reducing obesity among children and adolescents to 15.5% (7). Many health behaviors, deemed obesogenic behaviors, may contribute to the increasing rate of childhood obesity; these include the amount of physical activity and screen time children engage in and whether they receive the recommended amount of sleep (8–10). Food insecurity has also been associated with obesity from childhood into adulthood (11). Understanding these contributing factors to childhood obesity is imperative for improving efforts to reduce rates of obesity.

The proportion of US children who are overweight or obese is not evenly distributed. Rural children and adolescents are more likely to be overweight or obese than urban children and adolescents (12). Just under 20% of US children reside in rural areas, where the poverty rate is 25% higher than in urban areas (13). Higher poverty rates, which are associated with lower health literacy among rural children and their caregivers, may contribute to the overall poorer health status of rural children and adolescents (14,15).

More recent research examining rural–urban differences in overweight and obesity, physical activity, and food security among children and adolescents did not examine screen time or sleep guidelines (12). Yet, experiencing lower than recommended hours of sleep and higher than recommended hours of screen time is strongly associated with childhood obesity (9,16). Therefore, an updated and expanded study of obesogenic behaviors by rurality is needed. Previous studies on geographic differences in screen time and physical activity focused on populations outside the US (17), was limited to adults (18), was limited to 1 state (19), or used data that were more than a decade old (20). Therefore, an examination using updated national data of geographic differences in overweight and obesity, physical activity, food security, and recommended sleep and screen time guidelines met, is needed.

The objective of this study was to 1) examine rates of overweight and obesity, food security, and obesogenic behaviors, specifically physical activity, sleep time, and screen time, by rurality; and 2) examine the association between rurality and these 5 outcomes among a national sample of children and adolescents aged 10 to 17 years. We hypothesized that rural children and adolescents would have higher rates of all 5 outcomes, compared with their urban counterparts. The findings from this study should be useful for program developers as interventions are designed to improve health behaviors, particularly around sleep and sedentary behavior, in the US.

## Methods

We used data for this cross-sectional study from the 2021 and 2022 National Survey of Children’s Health (NSCH). The NSCH is a nationally representative survey sent online and via mail to adults aged 18 years or older who are caregivers of a child aged 0 to 17 years and randomly selected by the NSCH survey software. The NSCH includes questions about the health of the randomly selected child. The NSCH in 2021 and 2022 had 104,995 completed interviews, and results were weighted according to the population of US children.

### Study sample

We restricted our study to children and adolescents aged 10 to 17 years due to the suppression of weight information for children younger than 10 years in the NSCH; this restriction resulted in a sample size of 44,887. Furthermore, only participants that identified whether they lived in a rural or urban area were included, which reduced our sample size to 35,963. Children who lived in a metropolitan area (ie, area with a population of ≥50,000) were considered urban residents and those who did not live in a metropolitan area (ie, area with a population of <50,000) were considered rural residents, based on the rurality definition of the Office of Management and Budget. The NSCH does not release data on residence for states whose population of children is less than 100,000. The following states did not have data on residence in the 2021 and 2022 NSCH: Delaware, Maine, New Hampshire, North Dakota, South Dakota, Vermont, and Wyoming.

### Measures

The dependent variables of this study were body mass index (BMI), physical activity, food security, screen time, and hours of sleep. BMI was measured by using the caregiver’s recollection of the child’s height and weight and calculated by using the Centers for Disease Control and Prevention’s definitions: underweight, less than the 5th percentile; healthy weight, 5th percentile to less than the 85th percentile; or overweight or obese, 85th percentile or higher (21). Based on the US Department of Health and Human Services’ definition, the child was considered physically inactive if they did not participate in physical activity (eg, child exercises, sports) for at least 60 minutes every day (22). Participants were considered food secure if they could always afford nutritious meals, mildly food insecure if they could afford enough food but not always nutritious foods, and moderately to severely food insecure if they sometimes or often could not afford enough to eat. Screen time was categorized as less than 1 hour, 1 hour, 2 hours, 3 hours, or 4 or more hours per day. Hours of sleep was categorized as getting the recommended hours of sleep or not on an average weeknight based on the child’s age, which is 9 to 12 hours for children aged 10 to 12 years and 8 to 10 hours for adolescents aged 13 to 17 years.

The independent variables of this study consisted of variables related to the child and to the caregiver or household. The main independent variable of interest was rurality. The child variables were sex, age, race and ethnicity, and presence of special health care needs. The caregiver or household variables were the primary language of the household, highest level of educational attainment among adults in the household, family structure, and annual household income as a percentage of the federal poverty level (FPL). The presence of special health care needs was measured by using the Children with Special Health Care Needs Screener, a 5-item screening tool, based on the Maternal and Child Health Bureau definition and dichotomized as yes or no (23).

### Statistical analysis

Descriptive statistics were produced as percentages for the independent variables and dependent variables levels and compared between children living in rural areas and those living in urban areas by using Pearson χ^2^ and Cochran–Mantel–Haenszel tests. Multivariable logistic regression models examined the relationship between residence and the odds of being overweight or obese, physically inactive, food insecure, having more than 2 hours of screen time on the average day, and not meeting the recommended hours of sleep separately based on the independent variables (ie, sex, age, race and ethnicity, and presence of special health care needs). We used 2 hours or more of screen time as the cutoff because prior research recommended that children aged 5 to 17 years should be limited to no more than 2 hours of screen time per day (24,25). All analyses used the appropriate survey sampling weights, clusters, and strata provided by the NSCH. Data management and analysis were conducted in SAS version 9.4 (SAS Institute Inc). The University of South Carolina institutional review board deemed this study as exempt, nonhuman subjects research.

## Results

Of the children in the sample, 11.4% resided in a rural area, three-quarters (75.7%) were aged 10 to 15 years, and most (51.1%) were male (Table 1). Less than half (46.8%) were non-Hispanic White, more than a quarter (28.4%) were Hispanic, more than one-tenth (13.9%) were non-Hispanic Black, and a little more than one-tenth (10.9%) were another or combination of non-Hispanic race and ethnicity. Just over one-quarter (26.0%) of children had special health care needs. Less than sixteen percent (15.9%) came from households that did not speak English as their primary language, and most (69.2%) came from households where one of the caregivers or parents had at least some college education. Most children (59.7%) came from households that had 2 married parents and came from households with an income above 99% of the FPL (80.9%). Children residing in rural areas were more likely than children in urban areas to be non-Hispanic White and to have family structures other than 2 parents or a single caregiver.

**Table 1:** Characteristics of Children Aged 10 to 17 Years and Households, Overall (N = 35,963) and by Rural–Urban Residence, National Survey of Children’s Health, 2021–2022

Characteristic	All	Rural	Urban	*P* value^a^
	%			
**Overall**	100.0	11.4	88.6	—
**Child**				
**Sex**				
Male	51.1	51.6	51.0	.65
Female	48.9	48.4	49.0	
**Age, y**				
10–12	37.4	38.1	37.3	.11
13–15	38.3	39.6	38.1	
16–17	24.4	22.2	24.7	
**Race and ethnicity**				
Hispanic	28.4	14.9	30.1	<.001
Non-Hispanic Black	13.9	10.3	14.3	
Non-Hispanic White	46.8	67.4	44.2	
Non-Hispanic Other^b^	10.9	7.5	11.4	
**Has special health care needs**	26.0	27.6	25.8	.14
**Caregiver or household**				
**Primary language spoken in household is not English**	15.9	7.3	17.1	<.001
**Highest level of educational attainment of caregiver in the household**				
High school diploma or less/GED	30.8	36.7	30.0	<.001
Some college or more	69.2	63.3	70.0	
**Family structure**				
2 Parents, currently married	59.7	58.2	59.9	.005
2 Parents, not currently married	5.4	5.1	5.4	
Single caregiver household	26.5	25.7	26.6	
Other	8.4	11.0	8.1	
**Annual household income, % FPL**				
0–99	19.1	23.1	18.6	<.001
100–199	20.8	25.8	20.2	
200–399	29.0	30.9	28.7	
≥400	31.1	20.2	32.5	

Abbreviations: — , does not apply; FPL, federal poverty level; GED, General Educational Development.

a *P* values were calculated to compare rural–urban differences in respondent characteristics by using Pearson χ^2^ tests and Cochran–Mantel–Haenszel tests.

b Includes non-Hispanic Asian, American Indian or Alaska Native, Native Hawaiian or Other Pacific Islander, multiracial, and biracial.

Children residing in rural areas had a higher likelihood of being overweight or obese (38.3%) than children residing in urban areas (32.1%; *P* < *.*001) but a lower likelihood of being physically inactive (80.0%) than children residing in urban areas (85.3%; *P* < *.*001) (Table 2). Children residing in rural areas had a higher likelihood of being food insecure (40.1%) than children residing in urban areas (31.7%; *P* < *.*001) but a lower likelihood of having more than 2 hours of screen time on an average day (55.2%) than children residing in urban areas (60.5%; *P* = *.*001). Children residing in rural areas had a higher likelihood of not sleeping for the recommended age-appropriate hours during an average weeknight (39.7%) than children residing in urban areas (35.5%; *P* = *.*004).

**Table 2:** BMI Category, Physical Activity, Food Security, Screen Time, and Hours of Sleep, Children Aged 10 to 17 Years (N = 35,011), National Survey of Children’s Health, 2021–2022

Characteristic	All	Rural	Urban	*P* value^b^
	%			
**BMI^a^**				
Underweight	7.1	5.5	7.3	<.001
Healthy weight	60.1	56.2	60.6	
Overweight or obese	32.8	38.3	32.1	
**Physical activity**				
Inactive (less than every day of the week)	84.7	80.0	85.3	<.001
Active (60 minutes of physical activity every day)	15.3	20.0	14.7	
**Food security**				
Food secure	67.3	59.9	68.3	<.001
Food insecurity	32.7	40.1	31.7	
Mild food insecurity	27.3	32.3	26.7	
Moderate to severe food insecurity	5.4	7.8	5.0	
**Screen time, hours per day**				
<1	4.4	6.0	4.3	.001
1	9.9	10.2	9.9	
2	25.8	28.6	25.4	
3	23.9	21.8	24.2	
≥4	35.9	33.4	36.3	
**Child did not sleep for the recommended age-appropriate hours during an average weeknight**	36.0	39.7	35.5	.004

Abbreviations: BMI, body mass index.

a BMI is calculated as 1) underweight (BMI <5th percentile), 2) healthy weight (5th percentile to <85th percentile), and 3) overweight or obese (≥85th percentile); categories established by the Centers for Disease Control and Prevention.

b *P* values were calculated to compare rural–urban differences in respondent characteristics by using Pearson χ^2^ tests and Cochran–Mantel–Haenszel tests.

In multivariable logistic regression, rural children had higher odds of being overweight or obese (AOR = 1.36; 95% CI, 1.19–1.56), being food insecure (AOR = 1.27; 95% CI, 1.11–1.44), and not meeting the recommended hours of sleep (AOR = 1.23; 95% CI, 1.07–1.40) than urban children (Table 3). Rural children had lower odds of being physically inactive (AOR = 0.79; 95% CI, 0.70–0.90) and having more than 2 hours of screen time on an average day (AOR = 0.85; 95% CI, 0.75–0.96) than urban children. 

**Table 3:** Prediction of Overweight or Obesity, Physical Inactivity, Food Insecurity, More Than 2 Hours of Screen Time, and Not Meeting Recommended Sleep Hours, Children Aged 10 to 17 Years (N = 34,452), by Rural–Urban Residence, National Survey of Children’s Health, 2021–2022^a^

Characteristic	Model 1: overweight or obesity	Model 2: physical inactivity	Model 3: food insecurity	Model 4: >2 hours of screen time on an average day	Model 5: does not meet recommended hours of sleep
**Rurality**					
Rural	1.36 (1.19–1.56)^b^	0.79 (0.70–0.90)^b^	1.27 (1.11–1.44)^b^	0.85 (0.75–0.96)^b^	1.23 (1.07–1.40)^b^
Urban	1 [Reference]	1 [Reference]	1 [Reference]	1 [Reference]	1 [Reference]
**Child**					
**Sex**					
Male	1 [Reference]	1 [Reference]	1 [Reference]	1 [Reference]	1 [Reference]
Female	0.70 (0.63–0.77)^b^	1.80 (1.61–2.01)^b^	1.05 (0.94–1.16)	0.83 (0.76–0.91)^b^	1.00 (0.91–1.09)
**Age, y**					
10–12	1.31 (1.17–1.46)^b^	0.73 (0.64–0.83)^b^	0.95 (0.84–1.07)	0.55 (0.50–0.61)^b^	3.13 (2.81–3.49)^b^
13–15	1 [Reference]				
16–17	0.86 (0.76–0.97)^b^	1.00 (0.86–1.17)	0.99 (0.87–1.13)	1.18 (1.05–1.33)^b^	1.93 (1.71–2.17)^b^
**Race and ethnicity**					
Non-Hispanic White	1 [Reference]	1 [Reference]	1 [Reference]	1 [Reference]	1 [Reference]
Non-Hispanic Black	1.57 (1.36–1.82)^b^	1.34 (1.11–1.63)^b^	1.24 (1.06–1.45)^b^	1.40 (1.20–1.62)^b^	2.07 (1.79–2.38)^b^
Hispanic	1.72 (1.49–1.97)^b^	1.38 (1.16–1.64)^b^	1.37 (1.18–1.59)^b^	1.24 (1.08–1.41)^b^	1.13 (0.98–1.30)
Non-Hispanic Other^c^	1.07 (0.93–1.22)	1.23 (1.05–1.45)^b^	1.10 (0.95–1.27)	1.08 (0.95–1.22)	1.30 (1.15–1.47)^b^
**Has special health care needs**	1.42 (1.28–1.58)^b^	1.43 (1.26–1.62)^b^	1.60 (1.44–1.79)^b^	1.39 (1.25–1.53)^b^	1.21 (1.10–1.33)^b^
**Caregiver or household**					
**Primary language spoken in household is not English**	0.96 (0.79–1.16)	1.40 (1.09–1.79)^b^	0.72 (0.59–0.88)^b^	0.92 (0.77–1.11)	0.85 (0.71–1.03)
**Highest level of educational attainment of caregiver in the household**					
High school diploma or less/GED	1.45 (1.27–1.65)^b^	0.88 (0.76–1.01)	1.40 (1.23–1.59)^b^	0.99 (0.88–1.12)	1.16 (1.03–1.32)^b^
Some college or more	1 [Reference]	1 [Reference]	1 [Reference]	1 [Reference]	1 [Reference]
**Family structure**					
2 Parents, currently married	1 [Reference]	1 [Reference]	1 [Reference]	1 [Reference]	1 [Reference]
2 Parents, not currently married	1.47 (1.18–1.84)^b^	0.95 (0.74–1.23)	1.57 (1.26–1.96)^b^	1.28 (1.03–1.60)^b^	1.30 (1.05–1.61)^b^
Single caregiver household	1.13 (1.00–1.28)	1.04 (0.89–1.21)	1.42 (1.25–1.60)^b^	1.22 (1.09–1.37)^b^	1.29 (1.15–1.45)^b^
Other	0.93 (0.77–1.12)	1.05 (0.85–1.31)	1.11 (0.88–1.39)	1.20 (0.98–1.46)	1.29 (1.07–1.57)^b^
**Annual household income, % FPL**					
0–99	1.42 (1.21–1.67)^b^	0.75 (0.63–0.90)^b^	6.34 (5.35–7.52)^b^	0.88 (0.76–1.03)	1.27 (1.09–1.48)^b^
100–199	1.58 (1.36–1.82)^b^	0.79 (0.66–0.93)^b^	6.18 (5.31–7.20)^b^	1.04 (0.91–1.19)	1.34 (1.17–1.54)^b^
200–399	1.38 (1.24–1.55)^b^	1.07 (0.94–1.22)	3.38 (2.97–3.86)^b^	1.02 (0.92–1.13)	1.14 (1.02–1.28)^b^
≥400	1 [Reference]	1 [Reference]	1 [Reference]	1 [Reference]	1 [Reference]

Abbreviations: FPL, federal poverty level; GED, General Educational Development.

a All values are adjusted odds ratio (95% CI).

b Significant at *P* < .05.

c Includes non-Hispanic Asian, American Indian or Alaska Native, Native Hawaiian or Other Pacific Islander, multiracial, and biracial.

Female children were more likely than male children to be physically inactive (AOR = 1.80; 95% CI, 1.61–2.01) but less likely to be overweight or obese (AOR = 0.70; 95% CI, 0.63–0.77) and to have more than 2 hours of screen time on an average day (AOR = 0.83; 95% CI, 0.76–0.91). Children aged 10 to 12 years were more likely than children aged 13 to 15 years to be overweight or obese (AOR = 1.31; 95% CI, 1.17–1.46) and not meet the recommended hours of sleep (AOR = 3.13; 95% CI, 2.81–3.49). Race and ethnicity also had a significant effect: Hispanic children, compared with non-Hispanic White children, had a higher likelihood of being overweight or obese (AOR = 1.72; 95% CI, 1.49–1.97), being physically inactive (AOR = 1.38; 95% CI, 1.16–1.64), being food insecure (AOR = 1.37; 95% CI, 1.18–1.59), and having more than 2 hours of screen time on an average day (AOR = 1.24; 95% CI, 1.08–1.41).

Children with special health care needs, compared with children without special health care needs, were more likely to be overweight or obese (AOR = 1.42; 95% CI, 1.28–1.58), be physically inactive (AOR = 1.43; 95% CI, 1.26–1.62), be food insecure (AOR = 1.60; 95% CI, 1.44–1.79), have more than 2 hours of screen time on an average day (AOR = 1.39; 95% CI, 1.25–1.53), and not meet the recommended hours of sleep (AOR = 1.21; 95% CI, 1.10–1.33). Children whose caregivers or parents had not attained an education higher than high school or a GED, compared with children whose caregivers or parents had at least some college education, had higher odds of being overweight or obese (AOR = 1.45; 95% CI, 1.27–1.65), being food insecure (AOR = 1.40; 95% CI, 1.23–1.59), and not meeting the recommended hours of sleep (AOR = 1.16; 95% CI, 1.03–1.32).

Poverty also had a significant effect. Compared with children from a household with an income of 400% or above the FPL, children from a household with an income between 0% and 99% of the FPL were more likely to be obese or overweight (AOR = 1.42; 95% CI, 1.21–1.67), be food insecure (AOR = 6.34; 95% CI, 5.35–7.52), and not meet the recommended hours of sleep (AOR = 1.27; 95% CI, 1.09–1.48), but they had lower odds of being physically inactive (AOR = 0.75; 95% CI, 0.63–0.90).

## Discussion

This is the first study, to our knowledge, to examine geographic differences in the prevalence of overweight and obesity, food security, and obesogenic behaviors, specifically physical activity, sleep time, and screen time, using a national sample of children and adolescents. This research updates data on obesity, physical activity, and food insecurity and provides new information on sleep and screen time (12). Rural Healthy People 2030 recognized overweight and obesity as one of the top 5 priorities for improving rural health, while also ranking nutrition and physical activity among the top 10 focus areas (26). In both unadjusted and adjusted analyses, rural children were more likely than urban children to engage in physical activity and to have 2 hours or less screen time on an average day, which are positive behaviors; however, rural children were also more likely to experience food insecurity and to be overweight and obese, validating prior research (12). This study confirms earlier research using the NSCH finding that most children and adolescents in the US are meeting national guidelines for sleep, with fewer children and adolescents reaching the guidelines for physical activity and screen time (27).

A constellation of factors may contribute to more rural children being overweight or obese, compared with their urban counterparts, although we do not know the degree to which these are risk factors for obesity in a given setting. Rural children reside in poverty at much higher rates than urban children, with a much higher percentage of high-poverty counties in rural areas than urban areas (28). We found that children residing at or below the FPL had a higher likelihood than children residing 400% or above the FPL of being overweight or obese. Poverty has long been demonstrated to be associated with overweight or obesity status (29). The American Academy of Pediatrics has recommended screening for poverty and poverty-related social determinants of health, as these have been demonstrated to have negative consequences on child health and development (30).

Our results provide insight into the complexity of addressing childhood obesity. Previous research has consistently demonstrated a connection between obesogenic behaviors, such as physical inactivity and excessive screen time, with elevated BMI among children (31–33). However, our analysis suggests that the factors contributing to obesity may differ between urban and rural environments. In our analysis, compared with urban children, rural children had higher odds of being obese or overweight despite being less likely to be physically inactive or spend excessive time on screens. Although we found that rural children are more likely than urban children to be food insecure, a growing body of research suggests that food insecurity may result in increased consumption of ultra-processed calorie-dense foods (34,35). It is well established that energy imbalance — calorie intake exceeding expenditure — is the primary driver of weight gain and obesity (36). Although our data do not allow for precise measurement of calorie consumption, the energy imbalance model may help explain why, in our analysis, rural children were still more likely to be obese than urban children, despite being more physically active and having less screen time.

We found consistent sociodemographic disparities in our analyses. Compared with their non-Hispanic White counterparts, racial and ethnic minority and low-income children had significantly worse outcomes across obesogenic behaviors; they had higher odds of obesity, increased screen time, food insecurity, and physical inactivity. These disparities are in line with recent research that highlights the challenges faced by Black and Hispanic children, as well as those from lower-income households, who experience multiple barriers to achieving healthy behaviors and outcomes. These groups are more likely to live in environments with limited access to healthy food options, safe spaces for physical activity, and sufficient health care resources, which compound challenges in managing their weight and overall health (35).

The wide-ranging disparities observed in this study underscore the need for specialized interventions, particularly for rural children and for low-income children in racial and ethnic minority groups. Despite rural children being less likely to engage in excessive screen time or physical inactivity, they were still more likely to experience obesity, possibly due to factors like food insecurity and the consumption of calorie-dense, processed foods that are often more accessible and affordable in rural areas. The Institute of Medicine has recommended a need for multilevel interventions that focus on not just individual behaviors like physical activity and screen time but also broader environmental and systemic factors that influence children’s health outcomes (36).

### Strengths and limitations

Our study had multiple strengths. First, this is the most up-to-date analysis of rural and urban differences regarding weight, physical activity, food security, screen time, and hours of sleep. Second, the large sample size provided the statistical power to detect small differences. Third, the study used a national sample.

Our study also has limitations. The suppression of information from states with a population less than 100,000 children could leave some areas of the US underrepresented. Second, self-reported data present the potential for recall and social desirability bias, perhaps providing underestimates for health behaviors that could be perceived negatively. Third, the NSCH does not include children experiencing homelessness, so our analysis provides insight only into the health of children with a physical address.

### Conclusions

These findings are instructive for community partners and program developers in creating policies and programs that allow for multitiered interventions to promote positive health behaviors to reduce overweight and obesity among all children. The findings from this study can be used to reduce disparities in health behaviors between rural and urban children. Further research is needed to elucidate information on dietary intake among rural children, for which our study dataset did not allow.
